# Sensing of Co^2+^ and Cu^2+^ Ions Using Dimethylamino-functionalized Poly(azomethine-1,3,4-oxadiazole)s

**DOI:** 10.1007/s10895-024-03772-z

**Published:** 2024-06-05

**Authors:** Mihaela Homocianu, Elena Hamciuc, Corneliu Hamciuc

**Affiliations:** “Petru Poni” Institute of Macromolecular Chemistry, 41A, Grigore Ghica Voda Alley, 700487 Iasi, Romania

**Keywords:** Co^2+^ and Cu^2+^ ions, Polyazomethines, Absorption and fluorescence spectroscopy, Sensors

## Abstract

The ability of OxT and OxFl azomethines to recognize metal ions in THF solutions was investigated using UV-vis absorption techniques. Various metal ions, including Cd^2+^, Hg^2+^, Co^2+^, Sn^2+^, Cu^2+^, Ni^2+^, Zn^2+^ and Ag^+^, were tested. The absorption spectra revealed two distinct π-π* transition bands in the 273–278 nm and 330–346 nm wavelength ranges. Additionally, OxFl displayed an absorption peak at 309 nm, attributed to the fluorene group. Spectral titrations were used to study the fluorescence behavior in the presence of these metal ions. The results showed significant quenching with Co^2+^ and Cu^2+^ ions, while other metal ions had minimal effects on the fluorescence intensity. The quenching mechanism was further analyzed using the Stern-Volmer and Lehrer equations, and the binding constants (${\mathrm{K}}_{\mathrm{b}}^{\mathrm{fl}}$) were calculated using the Benesi-Hildebrand relations. The results confirm that Co^2+^ has a 1:2 stoichiometry and Cu^2+^ has a 1:1 stoichiometry, indicating the strong affinity of OxFl and OxT for these ions. The negative values of ΔG (Gibbs free energy) suggest that complex formation occurs spontaneously at room temperature.

**Supplementary Information:**

The online version contains supplementary material available at 10.1007/s10895-024-03772-z.

## Introduction

Poly(azomethine-1,3,4-oxadiazole) materials have potential applications in organic light-emitting diodes [1, 2], liquid crystals [3], and chemosensors due to their unique properties. These materials contain azomethine moieties that can form coordination bonds with metal ions. Materials containing 1,3,4-oxadiazole groups with N and O atoms are useful for chemosensing applications because they provide potential metal coordination sites.

Poly(azomethine-1,3,4-oxadiazole) materials are excellent for the detection of various cations, including Cu^2+^, [4, 5] Zn^2+^_,_ [6] Cd^2+^, [7] and Ag^+^, [8] due to the combination of azomethine and oxadiazole groups. Copper and cobalt ions are abundant transition metals in the human body and are involved in various physiological and biological processes. However, excessive levels of copper can lead to adverse health effects, including Alzheimer's disease, Parkinson's disease, Wilson's disease, and other pathological conditions [9, 10]. Therefore, it is crucial to detect and monitor these metal ions in real time to protect the environment and ensure human health and safety. According to the World Health Organization (WHO), the maximum levels of copper and cobalt ions in drinking water should not exceed 0.5 to 1 µM, and 31.4 µM, respectively [11, 12]. As a result, there is a growing need for efficient methods to detect and quantify these ions. Several techniques have been reported for detecting Co^2+^ and Cu^2+^ ions, including absorption spectrometry (AAS) [13, 14] inductively coupled plasma mass spectrometry (ICP-MS) [15], electrochemistry [16, 17], chemosensors [18–20], and voltammetry [21]. Optical sensors are the most suitable techniques due to their simplicity, low cost, and ease of use. Photoactive materials containing azomethine and 1,3,4-oxadiazole groups are promising candidates for the detection of metal ions because of their simplicity, low cost, high sensitivity, and ability to enable naked-eye detection. These materials have shown remarkable potential for the detection of Co^2+^ and Cu^2+^ ions. However, few studies have been published on the use of azomethine and 1,3,4-oxadiazole based photoactive materials for this purpose. Karami et al. [22] developed azomethine- AuNPs in an aqueous medium that can detect copper ions with a detection limit of 83.22 nM. Similarly, Divya and Thennarasu [23] synthesized an indole-pyrazole π-conjugate system for the colorimetric detection of micromolar Co^2+^ concentrations in environmental samples, which could be detected by the naked eye. New derivatives containing 1,3,4-oxadiazole groups have been prepared and used for the precise and sensitive fluorometric detection of Cu^2+^ ions [24, 25].

In our previous study [26], we analyzed the absorption and fluorescence spectral characteristics of OxFl and OxT in tetrahydrofuran (THF), dichloromethane (DCM), N-methyl-2-pyrrolidinone (NMP), and dimethyl sulphoxide (DMSO). The aim of this study was to evaluate the potential of OxFl and OxT as sensors for various metal ions, including Cd^2+^, Hg^2+^, Co^2+^, Sn^2+^, Cu^2+^, Ni^2+^, Zn^2+^, and Ag^+^. UV-Vis absorption and fluorescence titration experiments were performed. The experiments revealed distinct π-π* transition bands and significant fluorescence quenching upon additions of Co^2+^ and Cu^2+^ ions. These observations demonstrate the strong affinities of OxT and OxFl for these metals. The calculated binding constants and Gibbs free energy calculations suggest that the complex formation between these compounds and Co^2+^ and Cu^2+^ ions is spontaneous. These results demonstrate that OxFl and OxT can be used as effective sensors for the detection of Co^2+^ and Cu^2+^ ions in industrial water sources.

## Experimental Section

The OxT and OxFl polyazomethines were synthesized through solution polycondensation reactions. The reactions used a diamine and either terephthalic aldehyde or bis(4-formylphenoxyphenyl) fluorine as reactants, while NMP served as solvent [26].

### OxT

Yield 76%; FTIR (KBr, cm^−1^): 3040 (aromatic C-H), 2940 and 2880 (aliphatic), 1693 (terminal aldehyde group), 1620 (-CH = N- group), 1240 (aromatic ether linkage), 1019 and 960 (oxadiazole ring), 826 (p-substituted benzene); ^1^H NMR (400 MHz, CDCl_3_, ppm): 8.45 (**d**, 2H, J = 8.4 Hz, -CH = N- groups), 8.1–7.7 (**m**, C-H aromatic in ortho- position of -CH = N- units and 1,3,4-oxadiazole rings), 7.5–6.6 (**m**, aromatic), 5.60 (**s**, aliphatic CH of diamine segments), 3.05 (**s**, C-H of methyl from dimethylamino groups) [26].

### OxFl

Yield 71%; FTIR (KBr, cm^−1^): 3033 (aromatic C-H), 2930 and 2880 (aliphatic), 1694 (terminal aldehyde group), 1618 (-CH = N- group), 1240 (aromatic ether linkage), 1017 and 958 (oxadiazole ring), 826 (p-substituted benzene); ^1^H NMR (400 MHz, CDCl_3_, ppm): 8,56 (**d**, -CH = N- group)s, 8.1–7.8 (**m**, C-H aromatic in ortho- position of -CH = N- units and 1,3,4-oxadiazole rings), 7.6–6.6 (m, aromatic), 5.60 (**s**, aliphatic CH of diamine segments), 3.05 (**s**, C-H of methyl from dimethylamino groups) [26].

**Scheme 1:**
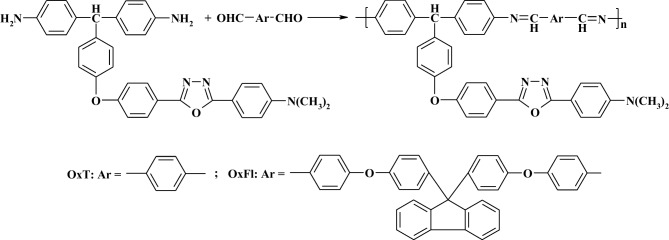
Molecular structures of OxT and OxFl compounds

The molecular structures of the OxT and OxFl polyazomethines investigated in this study are shown in Scheme 1. To evaluate their sensitivity to various metal ions, including M^n+^ (Cd^2+^, Hg^2+^, Co^2+^, Sn^2+^, Cu^2+^, Ni^2+^, Zn^2+^, and Ag^+^), a series of UV-Vis absorption and fluorescence experiments were performed. Spectral titrations were performed by incrementally adding M^n+^ (0–1500 μL, 10^–3^ M) to 2.5 mL solutions of OxT and OxFl (maintaining the concentration constant) in quartz cuvettes using a Hamilton syringe. Absorption spectra were recorded using a Shimadzu UV 3600 spectrophotometer, whereas fluorescence spectra were measured using a Perkin Elmer LS55 spectrofluorometer. Spectral profiles were recorded for each incremental addition of metal ions. The measurements were performed at room temperature using spectroscopic-grade solvents. The data were analyzed using Origin software.

## Results and Discussion

### Absorption Spectral Titrations

**Fig. 1:**
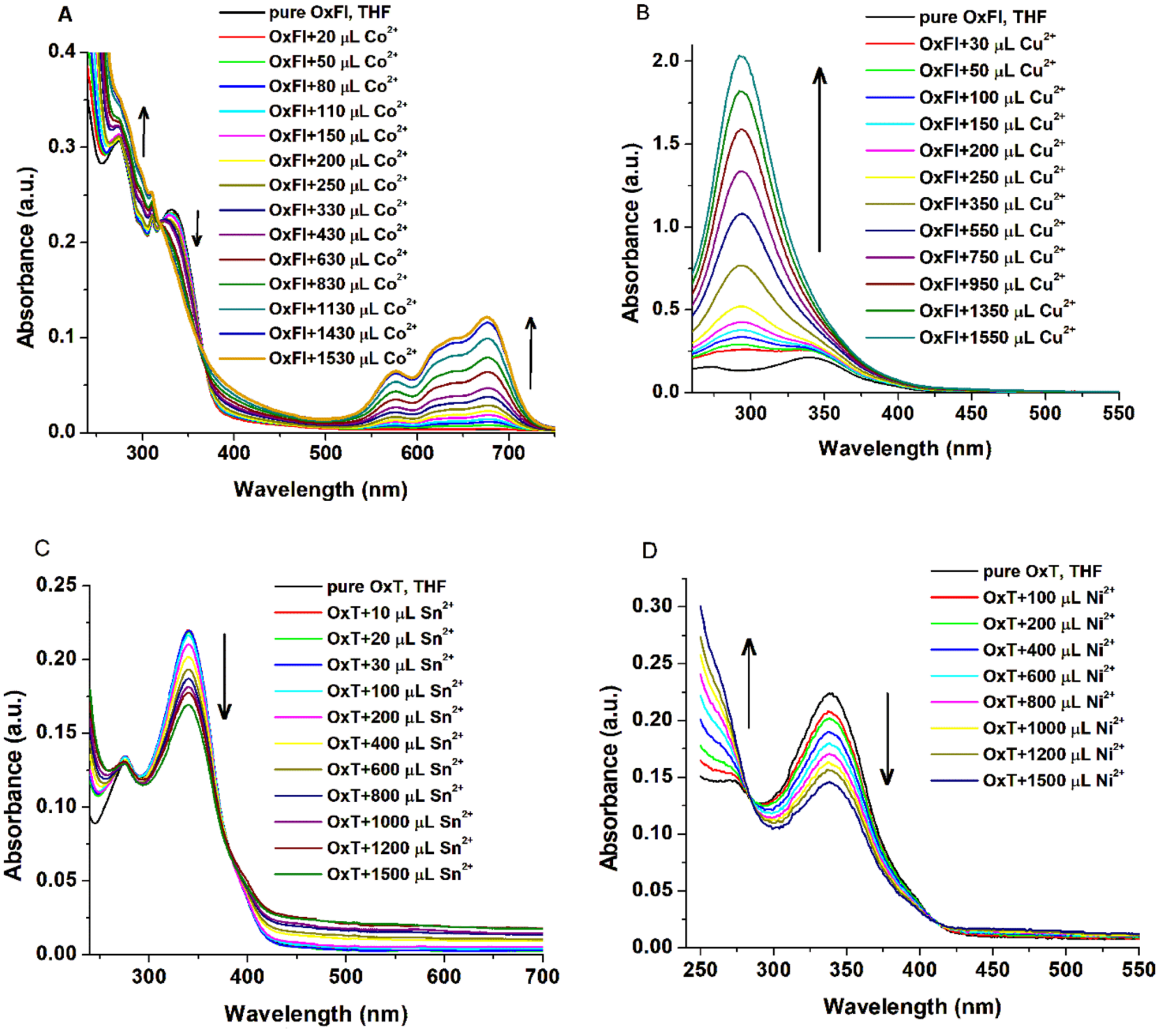
Absorption spectral changes of OxFl in THF solutions upon the addition of ~1500 µL of Co^2+^ (**A**), Cu^2+^ (**B**), and OxT upon the addition of ~1500 µL of Sn^2+^ (**C**), and Ni^2+^ (**D**) ions. The arrows in the figures indicate the variations in the absorption intensity corresponding to increasing metal ion concentrations

The ability of OxT and OxFl azomethines to detect Cd^2+^, Hg^2+^, Co^2+^, Sn^2+^, Cu^2+^, Ni^2+^, Zn^2+^, and Ag^+^ was investigated. The interactions between the polyazomethines and metal ions were characterized using UV-vis absorption spectroscopy. Figure 1 shows that both OxT and OxFl exhibit distinct π-π* transition absorption bands in the 273–278 nm and 330–346 nm wavelength regions. The absorption bands result from electronic transitions within the conjugated azomethine moieties of polyazomethines. The OxFl sample displayed an additional absorption peak at 309 nm, due to the fluorene moiety in the OxFl structure, which introduces a chromophore and affects the overall absorption profile [27].

The addition of metal ions caused significant changes in the absorption spectra of OxT and OxFl polyazomethines. The spectral changes were significant when titrated with increasing amounts of Co^2+^, Cu^2+^, Sn^2+^, and Ni^2+^ ions, showing the sensitivity of the polyazomethines to these metal ions (Fig. 1). In contrast, the presence of Cd^2+^, Hg^2+^, Sn^2+^, Ni^2+^, Zn^2+^, and Ag^+^ ions decreased the absorption intensity of the band centered at 302 nm for both azomethines (Supplementary Figs. S1 and S2). This indicates that the complexation of these metal ions with OxT and OxFl polyazomethines interrupts electronic transitions.

Interestingly, the addition of Cu^2+^ ions to the OxFl sample resulted in increased absorbance, suggesting the formation of a charge transfer complex between OxFl and Cu^2+^ ions. This demonstrates the selective recognition ability of OxFl for Cu^2+^ ions.

Furthermore, the introduction of Co^2+^ ions (Fig. 1A) to the OxFl and OxT solutions resulted in the appearance of a new absorption band with maxima at 676 nm and two additional shoulders at 576 and 632 nm, respectively. The spectral changes exhibited by these azomethines indicate their potential for selective identification and detection of metal ions.

### Fluorescence Spectral Titrations

**Fig. 2:**
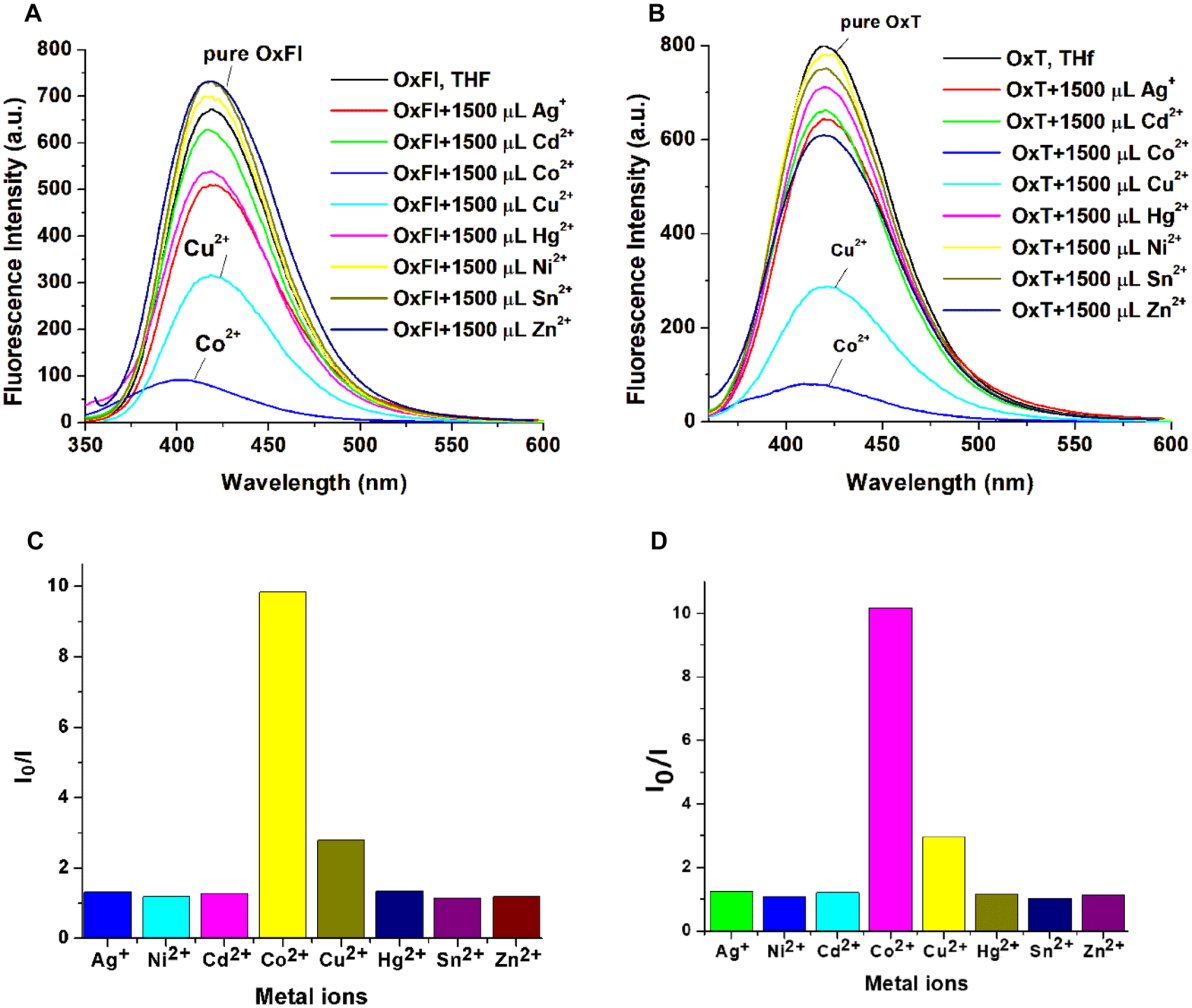
Fluorescence spectra (excitation wavelength - 300 nm) of pure OxFl (**A**) and OxT (**B**) and bar diagrams of I_0_/I for OxFl (**C**) and OxT (**D**) in THF solvent, created after the addition of 1500 µL of each metal ion

The fluorescence spectra of OxFl and OxT were recorded in THF solutions with varying concentrations of different metal ions, including Cd^2+^, Hg^2+^, Co^2+^, Sn^2+^, Cu^2+^, Ni^2+^, Zn^2+^, and Ag^+^. The emission peaks of pure OxFl and OxT were observed at 417 and 419 nm, respectively. Figure 2 shows that the fluorescence intensities of both compounds were significantly quenched upon the addition of 1500 µL of Co^2+^ and Cu^2+^ ions, whereas no significant changes were observed when equivalent amounts of other metal ions, such as Cd^2+^, Hg^2+^, Sn^2+^, Ni^2+^, Zn^2+^, and Ag^+^, were added. These variations in the fluorescence behavior can be attributed to the differences in the electronic properties, ionic radii, and chemical reactivity of Co^2+^ and Cu^2+^ compared to the other metal ions tested. The fluorescence intensity of the azomethine derivatives was quenched due to distinct binding modes or electron transfer reactions between Co^2+^ and Cu^2+^ ions and the azomethine moieties in OxFl and OxT. Two bar diagrams (Fig. 2C and D) were created to illustrate the changes in the fluorescence intensity caused by different metal ions. The diagrams show that fluorescence intensity decreases significantly with the addition of Co^2+^ and Cu^2+^, whereas other metal ions have minimal quenching effects.

**Fig. 3:**
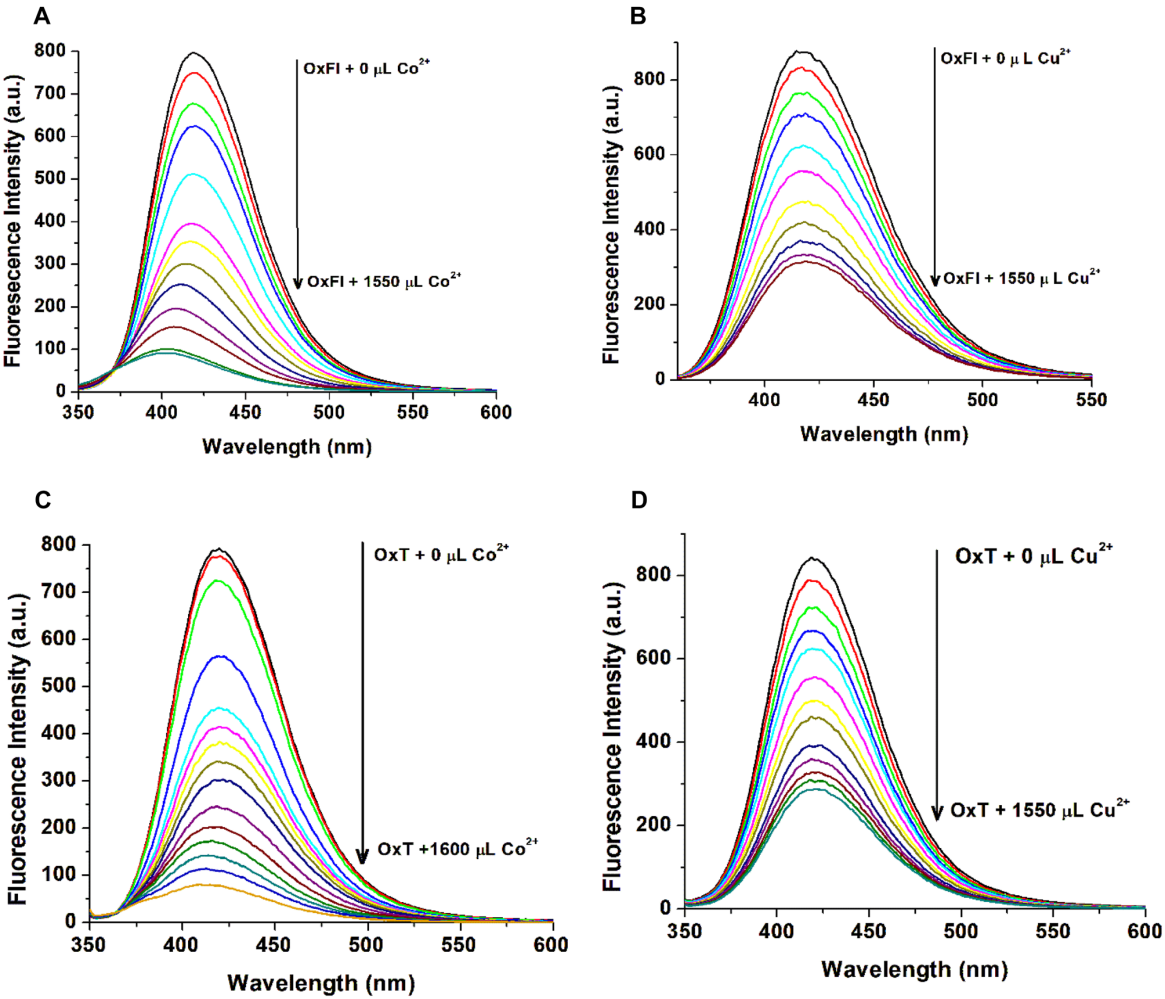
Spectrofluorometric titrations of OxFl and OxT azomethines (1.07 × 10^–3^ mol/L, λ_ex_ = 300 nm, λ_em_ = 417 nm) in THF solution with Co^2+^ (**A**, **C**) and Cu^2+^ (**B**, **D**) ions. The arrows show variations in emission intensity with increasing metal ion concentration

A detailed study investigated the effects of Co^2+^ and Cu^2+^ ion titrations on the OxFl and OxT solutions, as depicted in Fig. 3. The fluorescence spectra of pure azomethines in THF displayed intense emission bands, with maxima at 420 and 419 nm. As the concentration of Co^2+^ increased (Fig. 3A), the emission intensity decreased significantly, and the spectral band slightly blue-shifted from 410 to 404 nm, indicating the formation of the OxFl-Co^2+^ complex. However, when titrated with Cu^2+^ ions, the fluorescence intensity decreased without any significant spectral shift. Only Co^2+^ ions induced complete fluorescence quenching in both samples, resulting in an approximately 90% reduction in intensity (Fig. 3A and C). The quenched fluorescence reached a minimum plateau at approximately 1430 µL Co^2+^ for OxFl (Fig. 3A)*.* In contrast, titrations with Cu^2+^ resulted in a fluorescence intensity quenching of approximately 64.16% (Fig. 3B and D). Other tested metal ions, including Cd^2+^, Hg^2+^, Sn^2+^, Ni^2+^, Zn^2+^, and Ag^+^, had insignificant effects on fluorescence intensity, with the compounds remaining highly fluorescent (quenching percentage (QP %) was approximately 6.61%, as summarized in Table 1). The OxT sample exhibited a similar trend in the presence of Co^2+^ and Cu^2+^ (Fig. 3). These results demonstrate the high selectivity of OxFl and OxT azomethines for Co^2+^ ions.

**Table 1:** LOD, quenching percentage, quenching constant, and binding parameters for interactions of Co^2+^ and Cu^2+^ with OxFl and OxT polyazomethines

**Parameters**	**OxFl**		**OxT**	
	**Co**^**2+**^	**Cu**^**2+**^	**Co**^**2+**^	**Cu**^**2+**^
LOD (M)^a^	3.942 × 10^–5^	1.866 × 10^–5^	4.598 × 10^–5^	2.024 × 10^–5^
QP (%)^b^	89.84	64.16	90.08	66.09
Ksv (M^−1^, Eq. (1))^c^	1.42 × 10^4^	4.87 × 10^3^	1.54 × 10^4^	5.25 × 10^3^
K (M^−1^, Eq. (2))^d^	7.3 × 10^3^	-	4.45 × 10^3^	-
${\text{K}}_{\text{b}}^{\text{fl}}$(10^3^ M^−1^)^e^	2.01 × 10^8^	2.66 × 10^3^	4.99 × 10^9^	3.76 × 10^3^
ΔG (KJ mol^−1^)	-47.38	-19.54	-55.33	-20.39

^a^*LOD* limit of detection

^b^*QP* quenching percentage (QP = (I_0_-I)/I_0_ × 100%)

^c^*Ksv* quenching constant calculated with Stern-Volmer (SV) equation

^d^*K* quenching constant calculated with modified SV equation (Lehrer equation)

^e^ ${\text{K}}_{\text{b}}^{\text{fl}}$ Benesi-Hildebrand binding constant

The limit of detection (LOD) for Co^2+^ and Cu^2+^ ions using OxT and OxFl azomethines was determined based on fluorescence titration data. The LOD was calculated using the following relation: LOD = 3SD/slope. The “slope” was obtained from the linear plot between I_0_/I and [Co^2+^] or [Cu^2+^] concentrations (Fig. S3), and SD is the standard deviation of the blank signal. Table 1 summarizes the LOD obtained for copper and cobalt ions using OxFl and OxT azomethines. The calculated LOD values for Cu^2+^ using OxFl and OxT were approximately 1.866 × 10^–5^ and 2.024 × 10^–5^ M, respectively. These results indicate that the studied azomethines exhibited greater selectivity towards Co^2+^ ions than Cu^2+^. Literature reports the recognition of Cu^2+^ using different Schiff bases. The detection limits in this context range from 0.6–4.23 × 10^−6^ M [28, 29]. These findings offer valuable insights into the potential applications of OxFl and OxT compounds as selective fluorescent chemosensors for detecting cobalt and copper ions.

To evaluate and compare the sensitivity of OxFl and OxT to different metal ions, we used the Stern-Volmer (SV) equation (Eq. (1)) to evaluate the fluorescence quenching efficiency [30]. The SV equation describes the quenching data in a homogeneous environment and helps to elucidate the fluorescence quenching mechanism. However, for fluorophores in a heterogeneous environment, the modified Stern-Volmer equation, also known as the Lehrer equation (Eq. (2)), is more applicable [31]. This equation considers the possibility of quenching occurring through two mechanisms simultaneously: a combination of dynamic and static quenching, or the presence of a sphere of action [32].

The Stern-Volmer and Lehrer equations are expressed as follows:

1 $${\mathrm{I}}_{0}/\mathrm{I}=1+\mathrm{Ksv}[{\mathrm{M}}^{\mathrm{n}+}]$$

2 $${\mathrm{I}}_{0}/({\mathrm{I}}_{0}-\mathrm{I}) =1/\mathrm{f}+ 1/\mathrm{fK}[{\mathrm{M}}^{\mathrm{n}+}]$$

In these equations, I_0_ and I represent the fluorescence intensities in the absence and presence of the metal ions, respectively, [M^n+^] = [Cu^2+^, Co^2+^] denotes the metal ion concentration, and K_SV_ is the SV quenching constant, which indicates the accessibility of the azomethine molecules to the metal ions. To determine the y-intercept (f^−1^) and the slope (fK^−1^), I_0_/(I_0_-I) was plotted against 1/[M^n+^]. The modified SV quenching constant (K = Y-intercept/slope, Table 1) for the accessible population of azomethine molecules interacting with the metal ions can be calculated by dividing the y-intercept (f^−1^) by the slope (fK^−1^).

**Fig. 4:**
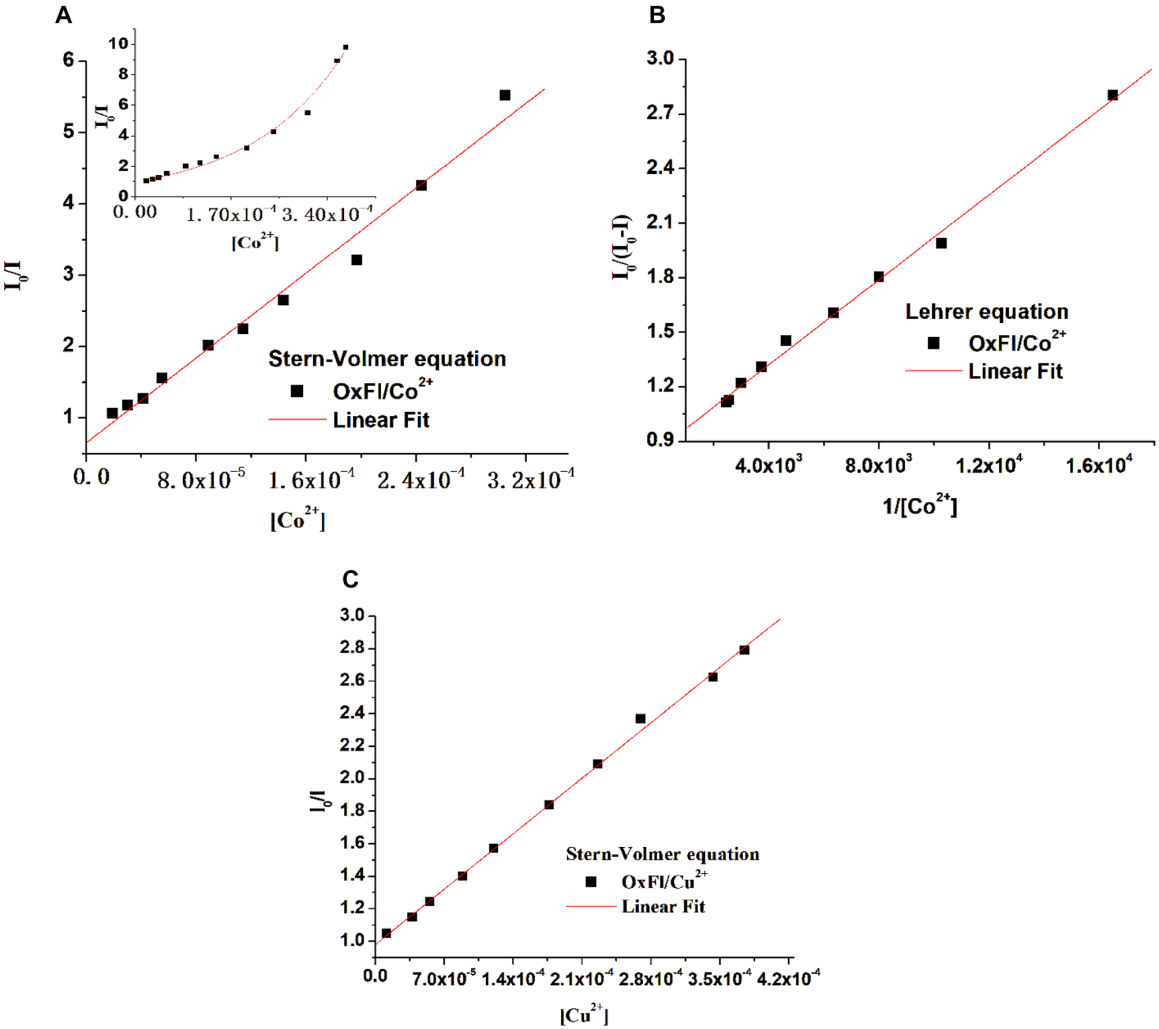
**A**, **C** Stern-Volmer and **B** modified Stern-Volmer plots (according to the Lehrer equation) for the fluorescence quenching of OxFl by Co^2+^ (**A**, **B**) and Cu^2+^ (**C**) ions. The inset displays the nonlinear plot of the Stern-Volmer curve for OxFl in the range of 0–3.4 × 10^–4^ mol/L Co^2+^

**Fig. 5:**
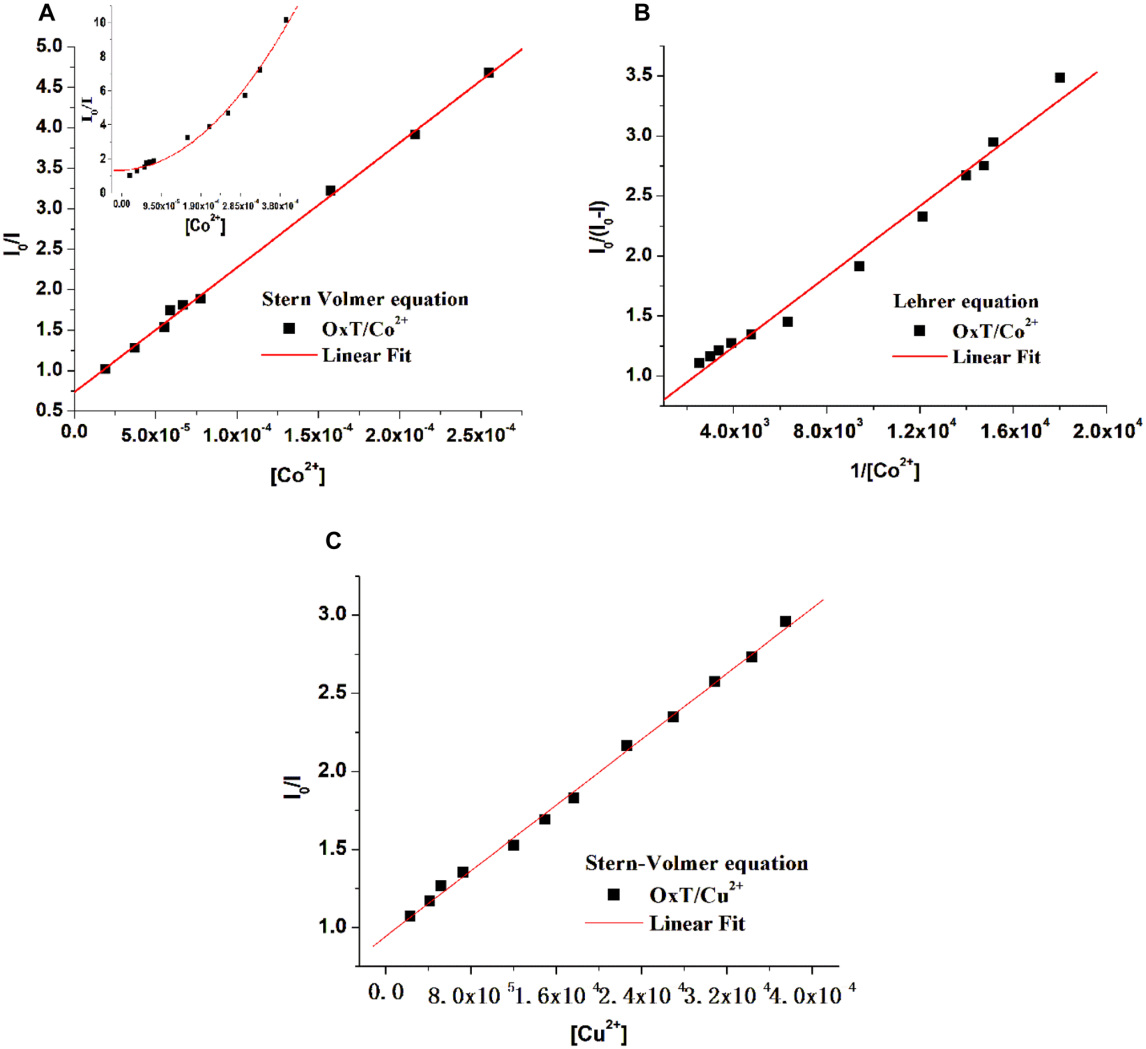
**A**, **C** Stern-Volmer and **B** modified Stern-Volmer plots (according to Lehrer equation) for the fluorescence quenching of OxT by Co^2+^ (**A**, **B**) and Cu^2+^ (**C**) ions. The inset displays the nonlinear plot of the Stern-Volmer curve for OxT in the range 0–3.8 × 10^–4^ mol/L Co^2+^

Figures 4A and 5A show that the Stern-Volmer plots for the OxFl and OxT compounds exhibited nonlinear behavior when titrated with Co^2+^ ions. This suggests that the quenching mechanism may involve a combination of static and dynamic processes. To gain a deeper understanding of the interaction mechanism, the experimental data were fitted to the Lehrer equation (a modified form of the SV equation, Eq. (2)), as illustrated in Fig. 4B. However, the SV plots became linear when titrated with Cu^2+^, as shown in Fig. 4C. When the results in Table 1 were analyzed, it was observed that the K_SV_ constants were greater than those obtained using the Lehrer equation. This observation suggests that the static process plays a more significant role in the quenching mechanism, despite the existence of dynamic quenching [33]. Furthermore, the addition of Cu^2+^ leads to a simpler fluorescence quenching mechanism for both OxFl (Fig. 4C) and OxT azomethines. The K_SV_ value was 1.42 × 10^4^ M^−1^ for OxFl/Co^2+^ and 4.87 × 10^3^ M^−1^ for OxFl/Cu^2+^ as shown in Table 1. These data indicate that the OxFl compound was more sensitive to Co^2+^ ions and had stronger interactions with cobalt ions (Fig. 5).

In conclusion, the use of Stern-Volmer and Lehrer equations allows the distinction between static and dynamic quenching processes. The results revealed that static quenching predominates when interacting with copper ions, while a combination of static and dynamic quenching was observed with cobalt ions. This distinction in quenching behavior highlights the sensitivity of OxFl and OxT compounds to specific metal ions. OxFl is more sensitive to Co^2+^ ions, indicating its potential for selective metal ion detection.

#### Determination of Binding Parameters

To better understand the binding affinity between OxFl and OxT and the metal ions Co^2+^ and Cu^2+^, we used the Benesi-Hildebrand relations (Eq. (3) [34, 35]) to calculate the binding constant (${\text{K}}_{\text{b}}^{\text{fl}}$)). The changes in the emission spectra associated with the formation of the respective complexes were analyzed using the following relations:

3 $$\frac{1}{{\mathrm{I}}_{0}-\mathrm{I}}=\frac{1}{{\text{I}}_{\mathrm{max}}-{\mathrm{I}}_{0}}+\frac{1}{{\mathrm{K}}_{\mathrm{b}}^{\mathrm{fl}}\left[{\mathrm{M}}^{\mathrm{n}+}\right]\left({\mathrm{I}}_{0}-{\mathrm{I}}_{\mathrm{max}}\right)}$$

where ΔI = I_0_-I represents the change in fluorescence intensity, ΔI_max_ = I_max_-I_0_ denotes the maximum change in fluorescence intensity, [M^n+^] is the concentration of the metal ion, and ${\text{K}}_{\text{b}}^{\text{fl}}$ is the binding constant.

**Fig. 6:**
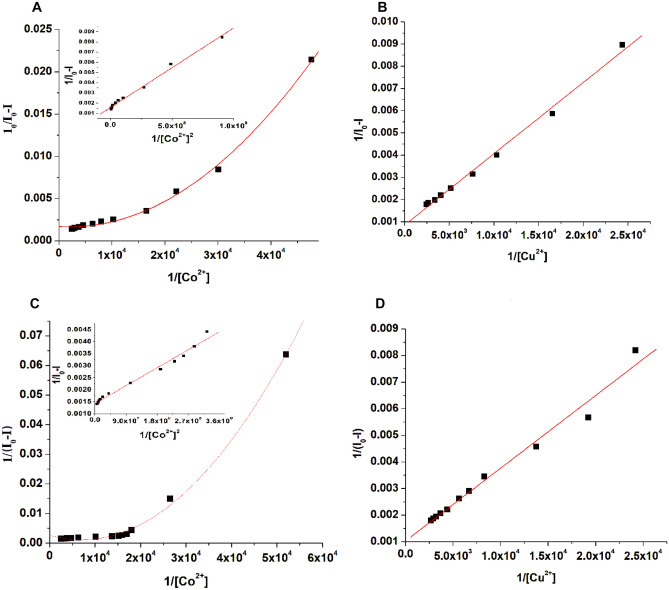
Benesi-Hildebrand plots for OxFl (**A**, **B**) and OxT (**C**, **D**) compounds with Co^2+^ (**A**, **C**) and Cu^2+^ (**B**, **D**) ions

The binding constants (${\text{K}}_{\text{b}}^{\text{fl}}$) for OxT and OxFl with Co^2+^ and Cu^2+^ ions were determined by analyzing the plot of 1/I_0_-I against 1/[Co^2+^] or 1/[Cu^2+^] (Fig. 6A–D). The results from Table 1 show that OxT has a higher binding constant of 4.99 × 10^9^ M^−1^, while OxFl has a lower binding constant of 2.01 × 10^8^ M^−1^ for binding with Co^2+^ ions. This suggests that OxT has a higher binding affinity for cobalt ions than OxFl. The binding constants indicated that OxFl and OxT bind strongly with Cu^2+^ ions in a 1:1 stoichiometry, as demonstrated by the linear plot of 1/I_0_-I *vs.* 1/[Cu^2+^] (Fig. 6B and D). In contrast, the complexation of Co^2+^ with OxFl and OxT azomethines has a 1:2 binding stoichiometry, confirmed by a polynomial curve, as shown in Fig. 6A and C. Table 1 summarizes the corresponding binding parameters for the fit. The study found that the OxFl and OxT azomethines have high affinities for Co^2+^ and Cu^2+^ ions, as determined by their binding constants.

The Gibbs free energy (ΔG) values for these processes were estimated using the following equation: ΔG = -2.303RTlog ${\text{K}}_{\text{b}}^{\text{fl}}$ [36], where ΔG (kJ mol^−1^) represents the free energy change of the complex, R is the universal gas constant (8.314 K^−1^ mol^−1^), ${\text{K}}_{\text{b}}^{\text{fl}}$ (M^−1^) is the binding constant obtained from the Benesi–Hildebrand plots and T (273 °C) is the absolute temperature in Kelvin. The calculated ∆G values are listed in Table 1. Negative values indicate that azomethines spontaneously form complexes with Co^2+^ or Cu^2+^ ions at room temperature, confirming their strong binding affinities for these ions.

The findings of this study (Table 1) were compared with the key features of other metal ion detection sensors published in the literature (Table 2).

**Table 2:** Key features of several metal ion detection sensors from the literature

**Metal ion** **detection sensors**	**Detection method**	**Metal ions detected**	**Sensitivity (limit of detection)**	**Selectivity**	**Response time**
Benzothiazole azo-derivatives [37]	Multi-sensing (colorimetric, fluorescence)	Cu^2+^ Co^2+^	Co^2+^ (6.4 × 10^−7^M) Cu^2+^ (8.4 × 10^−7^ M)	Good selectivity for Cu^2+^ and Co^2+^ Moderate (interference from other metal ions)	Rapid color change
Dihydropyridine [38]	Multi-sensing (colorimetric, fluorescence)	Cu^2+^ Co^2+^ Fe^3+^	Cu^2+^ (1.64 M) Co^2+^ (4.70 M)	Good selectivity for Cu^2+^, and moderate for Fe^3+^	Rapid color change
Donor-acceptor chalcone having phenanthrene and thiophene moieties [39]	Multi-sensing (colorimetric, fluorescence)	Co^2+^ Cd^2+^ Pb^2+^ Ni^2+^	-	Selective for Co^2+^	-
D-π-A Chalcone Analogue [40]	Fluorescence (turn-on-off-on)	Co^2+^,Fe^3+^ Na^+^_,_ K^+^	0.15 × 10^−3^ M	Selective for Co^2+^	Rapid fluorescence change
2-(2-hydroxyphenyl)-1-H-benzimidazole [41]	Fluorescence	Cu^2+^ Ru^3+^ Ni^2+^	Cu^2+^ (9.8 × 10^−4^ mol L^−1^) Ru^3+^ (7.2 × 10^−2^ mol L^−1^) Ni^2+^ (4.5 × 10^−3^ mol L^−1^)	-	-
Acid Red 94 [42]	-	Pb^2+^ Ag^+^	-	Selective for Pb^2+^ and Ag^+^	-

## Conclusions

In this study, the recognition ability of OxT and OxFl azomethines towards different metal ions in THF solution was investigated using UV-vis absorption and fluorescence spectroscopy. The absorption spectra of these compounds showed two distinct π-π* transition bands between 273–278 nm and 330–346 nm. OxFl exhibited an additional absorption peak at 309 nm due to the fluorene moiety. The presence of Co^2+^, Cu^2+^, Sn^2+^, and Ni^2+^ ions caused significant spectral changes, indicating the sensitivity of OxT and OxFl azomethines to these ions. Specifically, the fluorescence intensities of these compounds were significantly quenched in the presence of Co^2+^ and Cu^2+^, indicating their sensitivity to these specific ions. The fluorescence quenching mechanism was analyzed using the Stern-Volmer and Lehrer equations. The results indicate that static quenching was the main mechanism with Cu^2+^ ions, while a combination of static and dynamic quenching was observed with Co^2+^ ions. The binding constants indicate that OxT has a stronger binding affinity for Co^2+^ ions (4.99 × 10^9^ M^−1^) than OxFl (2.01 × 10^8^ M^−1^). Both azomethines demonstrated strong binding to Cu^2+^ ions. The negative Gibbs free energy values confirm that the azomethines bind strongly to Co^2+^ or Cu^2+^ ions and spontaneously form complexes at room temperature.

## Supplementary Information

Below is the link to the electronic supplementary material.

Supplementary file1 (DOCX 189 kb)

## Data Availability

No datasets were generated or analysed during the current study.
