# Supplementary material for: Sensing of Co2+ and Cu2+ Ions Using Dimethylamino-functionalized Poly(azomethine-1,3,4-oxadiazole)s
Source: J Fluoresc. 2024 Jun 5;35(5):3495–505. doi: 10.1007/s10895-024-03772-z (PMC12095355; doi:10.1007/s10895-024-03772-z)
Supplement: Supplementary file 1 — Supplementary file1 (DOCX 189 kb) [file 10895_2024_3772_MOESM1_ESM.docx]

**Supporting Information**

**For**

**Sensing of Co^2+^ and Cu^2+^ ions using dimethylamino-functionalized poly(azomethine-1,3,4-oxadiazole)s**

**Mihaela Homocianu^^[[1]](#footnote-1)^^, Elena Hamciuc, Corneliu Hamciuc**

*“Petru Poni” Institute of Macromolecular Chemistry, 41A, Grigore Ghica Voda Alley, 700487, Iasi, Romania*

|  |  |
| --- | --- |
|    |  |

**Fig. S1.** Absorption spectra of the OxFl sample in THF solution after the gradual additions of Cd^2+^ (A), Hg^2+^(B), Zn^2+^(C), Ag^+^ (D) and Mg2+ (E).

|  |  |
| --- | --- |
| **C**   | **D**  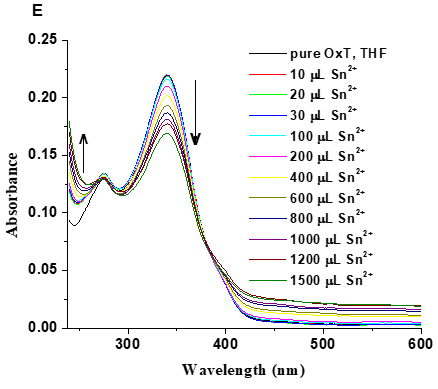 |

**Fig. S2.** Absorption spectra of the **OxT** sample in THF solution upon gradual additions of Cd^2+^ (A), Hg^2+^(B), Cu^2+^(C), and Sn^2+^(D).

|  |  |
| --- | --- |
|  |  |

**Fig. S3.** LOD plots of OxFl and OxT compounds for the detection of Co^2+^ and Cu^2+^ ions, at 417 and 419 nm, respectively.

1. Corresponding author: Tel.: 0232 217 454.

   *E-mail address*: [michalupu@yahoo.co.uk](mailto:michalupu@yahoo.co.uk) and [mlupu@icmpp.ro](mailto:mlupu@icmpp.ro) (Mihaela Homocianu). [↑](#footnote-ref-1)
